# A Repeated Time-to-Positive Symptoms Improvement among Malaysian Patients with Schizophrenia Spectrum Disorders Treated with Clozapine

**DOI:** 10.3390/pharmaceutics13081121

**Published:** 2021-07-22

**Authors:** Orwa Albitar, Sabariah Noor Harun, Siti Nor Aizah Ahmad, Siti Maisharah Sheikh Ghadzi

**Affiliations:** 1 School of Pharmaceutical Sciences, Universiti Sains Malaysia, Gelugor 11800, Malaysia; sabariahnoor@usm.my; 2 Psychiatric Department, Hospital Pulau Pinang, Ministry of Health Malaysia, Jalan Residensi, George Town 10460, Malaysia; siti_naa@yahoo.co.uk

**Keywords:** clozapine, repeated time-to-event, hazard, NONMEM, positive symptoms

## Abstract

Clozapine remains the drug of choice for resistant schizophrenia. However, its dose-response relationship is still controversial. The current investigation aimed to develop a repeated time-to-positive symptoms improvement following the onset of clozapine treatment in Malaysian schizophrenia spectrum disorder patients. Data from patients’ medical records in the Psychiatric Clinic, Penang General Hospital, were retrospectively analyzed. Several parametric survival models were evaluated using nonlinear mixed-effect modeling software (NONMEM 7.3.0). Kaplan–Meier-visual predictive check (KM-VPC) and sampling-importance resampling (SIR) methods were used to validate the final model. A total of 116 patients were included in the study, with a mean follow-up of 306 weeks. Weibull hazard function best fitted the data. The hazard of positive symptoms improvement decreased 4% for every one-year increase in age over the median of 41 years (adjusted hazard ratio (aHR), 0.96; 95% confidence intervals (95% CI), (0.93–0.98)). However, patients receiving a second atypical antipsychotic agent had four-folds higher hazard (aHR, 4.01; 95% CI, (1.97–7.17)). The hazard increased 2% (aHR, 1.02; 95% CI, (1.01–1.03)) for every 1 g increase in the clozapine six months cumulative dose over the median of 34 g. The developed model provides essential information on the hazard of positive symptoms improvement after the first clozapine dose administration, including modifiable predictors of high clinical importance.

## 1. Introduction

Schizophrenia spectrum and other psychotic disorders (SSD) are serious and chronic illnesses characterized by positive symptoms such as hallucinations, delusions, abnormal motor behavior, disorganized thinking, and negative symptoms such as a diminished emotional expression or avolition [1]. The number of SSD patients in Malaysia was 15,104 in 2015, with an economic burden of USD 100 million [2].

Treatment-resistant schizophrenia (TRS) is defined as persistent refractory symptoms after at least two different classes of antipsychotic treatments with an optimized dose for more than six weeks [3]. More than 30% of SSD patients were reported to encounter TRS [4]. The economic burden of TRS treatment was shown to be 3–11 folds higher than non-TRS schizophrenia [5]. When residual symptoms persist and antipsychotic monotherapy has already been provided with sufficient dose and duration, only a few treatment choices remain [3].

Despite its side effects [6], clozapine (CLZ) remains the treatment of choice for unresponsive patients to a minimum of two trials of antipsychotic drugs [7]; it is also recommended particularly for refractory patients or those intolerant to the side effects of first-generation antipsychotics such as chlorpromazine and haloperidol [8]. Furthermore, CLZ is indicated to reduce the suicide risk in patients with a history of schizophrenia [9]. In a recent meta-analysis, SSD patients treated with CLZ had lower all-cause discontinuation and re-hospitalization and better outcomes regarding overall symptoms compared with other second-generation antipsychotics [10].

In a population pharmacodynamic and pharmacokinetic model (PKPD) among schizophrenia patients, total daily doses of 250–500 mg were required to significantly affect positive and negative symptoms scale (PANSS) within 12 weeks of clozapine treatment. Furthermore, different doses were recommended based on sex and smoking due to their effect on clozapine pharmacokinetics [11,12]. The current study aimed to develop a repeated time-to-event model of SSD positive symptoms improvement in Malaysian patients with a long follow-up period and investigate relevant demographic and clinical characteristics as potential covariates, including the drug exposure in terms of the total daily dose, frequency of administration as well as concomitant medications.

## 2. Materials and Methods

### 2.1. Study Design

A retrospective study design was adapted to obtain data from SSD patients’ medical records from the Psychiatric Clinic, Penang General Hospital, Malaysia. All SSD patients from 1999 to 2020 that received clozapine were included, while those who did not receive clozapine or received it for purposes other than SSD were excluded. Drug doses, frequency, other clinical and demographic data were extracted from patients’ files in the psychiatric clinic. This study was approved by the Medical Research and Ethics Committee, Ministry of Health, Malaysia (Research ID: NMRR-19-1297-46641) on 5th December 2019.

### 2.2. Data Collection

Patient’s demographic data, e.g., age, sex, race, smoking, and body mass index (BMI), as well as relevant clinical data such as total daily dose, dosing frequency, blood pressure, heart rate, white blood cells count, hemoglobin levels, platelets count, absolute neutrophils count, and concomitant medications. Positive symptoms were defined as the presence of hallucinations, delusions, abnormal motor behavior, or disorganized thinking based on the Diagnostic and Statistical Manual of Mental Disorders, Fifth Edition (DSM-V). Symptoms were recorded on each follow-up by the psychiatrist specialist.

### 2.3. Model Development

A repeated time-to-event model was developed to evaluate the improvement of the positive symptoms following the onset of clozapine treatment. Symptoms improvement was defined as the absence of positive symptoms following existing, occasional, on and off, or residual positive symptoms. The last observation time (end of follow-up) was recorded and treated as a censored observation. The model development process involved two steps: (i) base model development without any explanatory factors apart from time; and (ii) exploration of potential covariates to develop the full model. The model structure and its parameters were estimated using the Laplace estimation method through the nonlinear mixed-effect modeling software, NONMEM 7.3.0, (ICON Clinical Research LLC, Ellicott City, MD, USA) [13].

### 2.4. Base Model

A parametric survival function was implemented to describe the SSD positive symptoms in a repeated time-to-event model, according to Equation (1):

(1) $$S\left(t\right)=e^{-\int_{0}^{t}h\left(t\right)dt}$$

where *h*(*t*) is the hazard, and *S*(*t*) is the survival, which is a function of the cumulative hazard within the time interval between zero (clozapine first dose) and the time t describing the probability of not experiencing positive symptoms improvement within this interval. Random effects on the hazard function were incorporated as symptoms improvement can be experienced again after symptoms deterioration in multiple observations per subject; therefore, inter-individual variability in baseline hazard was described in an exponential error model based on the following formula:

(2) $$\theta_{i}=\theta \times e^{\eta_{i}}$$

where *θ_i_* is the individual estimation of hazard function parameters, *θ* is the estimated hazard function parameters of the population, and *η_i_* is the inter-individual random effects that are assumed to be randomly distributed with a mean of 0 and variance of ω^2^.

Different functions for the hazard were explored to develop the base model, starting from a simple time-independent constant hazard based on the following equation:

(3) $$h=\lambda_{0}\times e^{0}$$

more complex functions such as Gompertz and Weibull hazard functions [14] were also investigated according to Equations (4) and (5), respectively:

(4) $$h\left(t\right)=\lambda_{0}\times e^{\beta \times t}$$

(5) $$h\left(t\right)=\lambda_{0}\times e^{\beta \times \ln\left(t\right)}$$

while both functions assume a varying hazard over time, the Weibull function requires some attention as it assumes a hazard equal to zero when time is zero [15].

The likelihood ratio test was used to discriminate between nested models with a significance level (α) of 5% (equivalent to a reduction in objective function value (OFV) of 3.84 for one degree of freedom and 5.99 for two degrees of freedom).

### 2.5. Full Covariate Model

The potential covariates such as demographic and clinical characteristics were explored with the best-developed base model. The total daily dose and the cumulative dose were evaluated in different parameterizations, including Emax models. Continuous and categorical covariates were added based on the exponential model as in Equations (6) and (7), respectively, where continuous covariates were centered on the median covariate value:

(6) $$h\left(t\right)=\theta_{1}\times e^{\left(\theta_{2}\times \ln\left(t\right)+\theta_{cov}\left(cov_{i}-cov_{median}\right)\right)}$$

(7) $$h\left(t\right)=\theta_{1}\times e^{\left(\theta_{2}\times \ln\left(t\right)+\theta_{cov}\left(cov_{i}\right)\right)}$$

*θ*_1_ and *θ*_2_ are scale and shape parameters of the Weibull hazard function, respectively, *cov_i_* is the respective covariate value for the individual, and *θ_cov_* is the covariate coefficient.

Univariate analysis was performed initially to evaluate the relationship between each covariate and the base hazard separately. Then significant factors were further evaluated in a stepwise forward addition procedure (*p* < 0.05) equivalent to a reduction of 3.84 in OFV, followed by a backward elimination procedure (*p* < 0.01) equivalent to a decrease of 6.64 in OFV.

### 2.6. Model Evaluation and Validation

The final model was validated to ensure that it describes the data sufficiently. This was done through a visual predictive check (VPC) [16] as implemented in PsN (version 4.9.0, Uppsala, Sweden) with 1000 simulations to perform Kaplan–Meier-VPC (KM-VPC) plot. In order to perform simulations for time points with no clinical observations, extra data records were added to the dataset every week for 800 weeks. The covariates for these additional data records were linearly interpolated between the original records. The model performance was also evaluated through relative standard error (RSE) produced from the SIR method using 1000 samples [17]. The final model was chosen based on the lowest OFV, diagnostic plots (e.g., KM-VPC), and scientific plausibility. The data and results were visualized using Xpose4 (version 4.6.1, Uppsala, Sweden) [18,19,20].

## 3. Results

### 3.1. Patients’ Demographics and Clinical Data

A total of 3098 follow-up points were traced for 116 SSD patients. The demographic and clinical characteristics of the patients are represented in Table 1.

The majority of the patients were Malaysian Chinese (74, 63.8%), females (65, 56.0%), having a mean (range) BMI of 25.4 Kg · m^−2^ (13.3–46.1) and age 41 years (9.2–78.4). Sixty-two (53.4%) of the patients had shown improving symptoms; symptoms improvement was recorded on 122 occasions (average two per patient). All patients have received a mean (range) total daily dose of clozapine, 254.2 mg (6.25–825) as a single agent or in combination with typical or atypical antipsychotic agents. Patients were followed up (censored) for a mean (range) of 306 weeks (8–800), where symptoms improvement was still recorded after a mean [range] of 226 weeks (0–778).

### 3.2. Base Model

**Figure 1:**
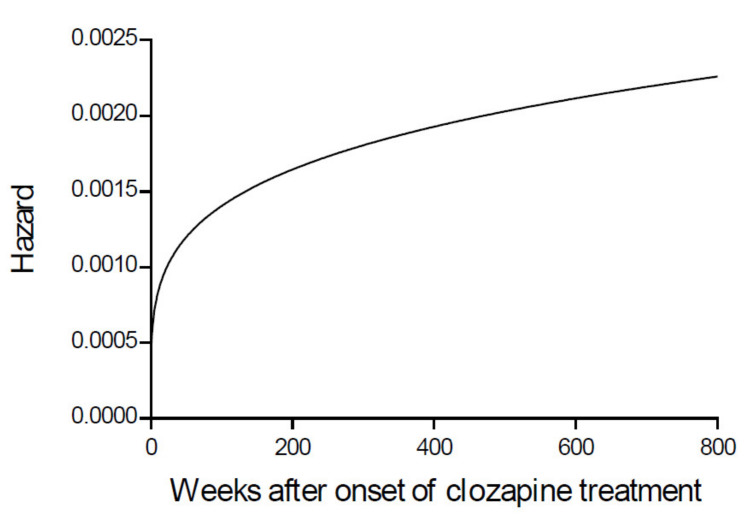
Weibull base model illustrating the shape of improving positive symptoms hazard function in a time window of 800 weeks after the onset of clozapine treatment. The hazard is near zero at time zero, then exponentially increases following clozapine treatment.

Table 2 shows the OFVs of the investigated different functions. The Weibull hazard function gave the best result regarding OFV, KM-VPC plots, and RSE computed from SIR. The model represents a very sharp increase in the hazard of improved positive symptoms during the initial 24 weeks of clozapine treatment achieving almost 50% of the maximum recorded hazard, as elaborated in Figure 1.

### 3.3. Covariate Model

Univariate analyses of the covariates are summarized in Table 3. Older age and concomitant administration of statins were associated with a lower hazard of positive symptoms improvement while receiving a second atypical antipsychotic agent, and higher clozapine cumulative doses achieved after six months of treatment resulted in a higher hazard of positive symptoms improvement. Other covariates such as clozapine total daily dose, sex, race, smoking, and other concomitant medications had no significant impact on positive symptoms hazard.

Significant covariates were included in the model building. Age, concomitant administration of a second atypical antipsychotic agent, and cumulative clozapine dose achieved after six months were retained after forward inclusion, and finally, none of the included covariates was removed during backward elimination as shown in Table 4. Based on the final model, the hazard of SSD positive symptoms improvement decreases around 4% for every one-year increase in age over the median of 41 years (adjusted hazard ratio (aHR), 0.96; 95% confidence intervals (95% CI), (0.93–0.98)). However, the hazard increased by four-folds (aHR, 4.01; 95% CI, (1.97–7.17)) in patients receiving a second atypical antipsychotic agent. The hazard of improving positive symptoms increased 2% (aHR, 1.02; 95% CI, (1.01–1.03)) for every 1 g increase in the clozapine cumulative dose achieved after six months over the median of 34 g (control stream and simulated data are in the Supplementary Materials).

### 3.4. Model Validation

**Figure 2:**
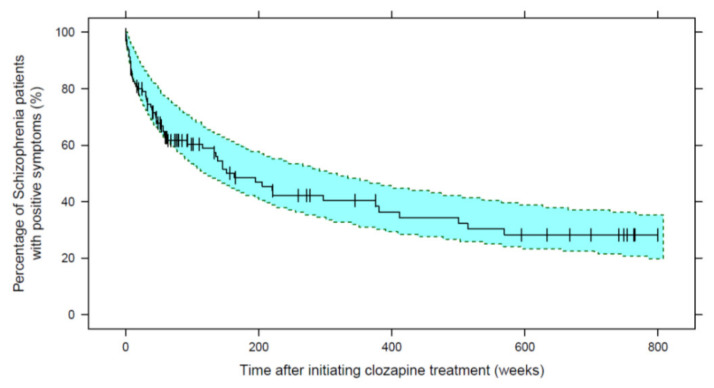
The final repeated time-to-event model of improving positive symptoms after the onset of clozapine treatment. The solid line represents the observed Kaplan–Meier survival plot while vertical lines mark censored observations with a mean time, (range) of 306 weeks (8–800). Shaded areas represent the 95% prediction interval from 1000 simulated datasets.

**Figure 3:**
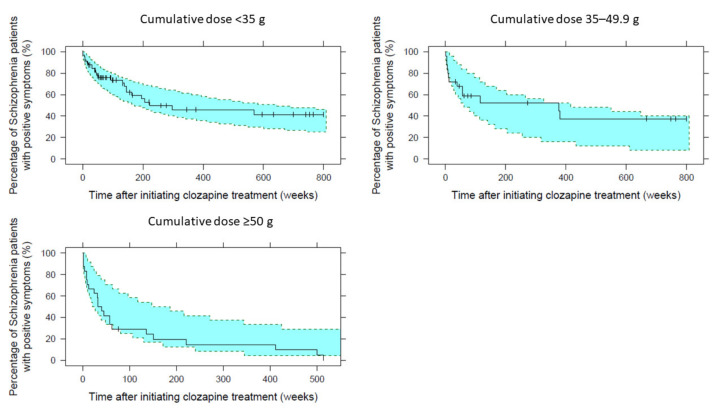
The final repeated time-to-event model of improving positive symptoms after the onset of clozapine treatment stratified on cumulative dose achieved after six months groups. The solid line represents the observed Kaplan–Meier survival plot while vertical lines mark censored observations with a mean time, (range) of 306 weeks (8–800). Shaded areas represent the 95% prediction interval from 1000 simulated datasets.

Based on the KM-VPC plots, the 95% confidence interval of the simulated data overlaid the observed data, implying the good predictive performance of the final model, as seen in Figure 2. The models’ parameters have shown RSEs of less than 36% based on the SIR results, as shown in Table 5. Furthermore, Figure 3 represents the final model stratified based on cumulative dose groups, where those with cumulative doses more or equal than 50 g after the initial six months of treatment have all demonstrated improvement before 500 weeks. Stratification-based concomitant APA administration (Figure S1) and age groups (Figure S2) can be found in the Supplementary Materials.

## 4. Discussion

A repeated time-to-event model for positive symptoms improvement after the onset of clozapine treatment is introduced in the current study. The observed data with a long mean follow-up period of 306 weeks from 116 SSD patients were adequately described in a Weibull hazard function. Among the 17 investigated potential covariates, age, concomitant treatment with a second antipsychotic agent, and higher clozapine cumulative doses achieved after six months of treatment had a significant statistical effect.

Parametric modeling is a practical and powerful approach that assumes a specific survival time distribution [21]. Compared to nonparametric and semi-parametric methods such as Kaplan–Meier analysis and Cox proportional hazard model, the parametric modeling enables the evaluation of time-varying covariates that may influence each other taking into account censoring as well as the simulation of time-to-event data according to the final model [22,23].

The current analysis reveals a very low hazard of improving positive symptoms (near zero) on the day of clozapine treatment initiation. This is followed by an exponential increase in the hazard of symptoms improvement weeks after the clozapine treatment representing the typical shape of the Weibull hazard function. In earlier studies among TRS patients, it was recommended that the clozapine treatment be discontinued if response was not achieved within eight weeks of clozapine treatment [24], despite the reported low response rate of around 30% by week 8 [25,26,27]. A few studies also reported improving clozapine response rate for up to 50% by week 12 [25,28,29], while it took 24 weeks for 71.9% of the patients to achieve the response criteria [25]. A consensus guideline for reporting and determining sufficient treatment response was introduced by the Treatment Response and Resistance in Psychosis (TRRIP) Working Group [8]. It was recommended in the TRRIP guideline that the clozapine treatment should be maintained for at least three months after achieving the therapeutic range before evaluating patients as non-responsive [8,30]. In agreement with the previous studies, the current study showed that the time course of positive symptoms improvement after initiating the clozapine treatment is not constant over time and may have delay onset up to 24 weeks or require time to achieve a ‘steady state’ before the maximum effect can be observed. Future studies may be required to determine the extent of SSD progression/changes over time that may affect the improvement of positive symptoms.

In terms of the clozapine dose, it was shown in a PKPD model in schizophrenia patients that a total daily dose of 250–500 mg was shown to achieve 50% improvement in PANSS within 12 weeks of treatment [11]. In a meta-analysis of five short to moderate term trials (6–16 weeks) trials, a standard daily dose of 300–600 mg was not shown to be superior as compared to low (150–300 mg/day) and very low doses (<150 mg/day) [31], which was consistent with the current study results where the total daily dose was not shown to be a significant covariate on the hazard of improving positive symptoms. However, the total cumulative dose of clozapine achieved after six months of treatment was significantly describing the exposure to the drug, taking into account both the dose and time. Recently, higher clozapine doses, concentrations, and treatment durations were reported in responders as compared to non-responders [32]. Furthermore, waiting for a delayed clozapine response for persistent positive symptoms was recommended over plasma level-guided dose escalation in the recent consensus recommendations by the TRRIP working group [33].

In a recent meta-analysis on SSD patients on clozapine, younger age was the only reported demographic predictor for clozapine response [34], consistent with the current results. Younger patients might have lower disease severity and duration regardless of the type of treatment they are receiving [34,35]; thus, it was recommended not to wait too long before initiating clozapine treatment [34]. In another systematic review of randomized control trials, age did not achieve statistical significance [36]. However, the included studies in the second review were limited to adult patients with ages ranged between 21 and 65 years old.

Prescribing a combination of antipsychotic agents is a common clinical practice to improve patients response [37]. In a systematic review of antipsychotic combinations for schizophrenia, combinations that especially included clozapine and typical antipsychotics demonstrated preferable outcomes compared to the monotherapy or atypical antipsychotics combinations [38]. Another meta-analysis revealed that atypical antipsychotics combined with clozapine did not improve positive symptoms despite positively affecting negative and depressive symptoms [39]. However, in a recent multicenter investigation with long follow-up, clozapine augmentation with the atypical antipsychotic, paliperidone, has shown significant relief of symptoms, lower number of hospitalization, and improved functionality [40]. Finally, a consensus of treatment strategies for refractory schizophrenia patients was introduced by the TRRIP working group, where combinations of clozapine and atypical antipsychotics such as aripiprazole or amisulpride were recommended for refractory positive and mixed symptoms [33], in line with the current study findings.

Add-on statins therapy was also a subject for investigation in a meta-analysis including five studies in patients receiving antipsychotic agents. Statin therapy had resulted in a preferable outcome in terms of total PANSS scores without significant change in the subscale PANSS. However, results were limited by the small sample size of 236 patients [41]. Furthermore, they might not apply to all types of antipsychotics, particularly clozapine, where relapse was reported in one patient on clozapine shortly after he received atorvastatin, while improvement of symptoms was observed once atorvastatin was withheld [42]. This was consistent with the current univariate evaluation of statins treatment. A possible explanation would be the potential binding of clozapine with plasma lipoproteins [43], which might have a role in drug delivery to the site of action and potentially be altered with statin treatment [44].

Bodyweight was not successfully included as a factor predicting clozapine response in a recent meta-analysis [34]. However, in two recent investigations, BMI increase predicted an improved outcome in terms of both positive and negative symptoms [45,46], which was inconsistent with the current findings. Weight gain might be a possible side effect resulting from clozapine treatment rather than reflecting an improved response [47]. Other demographic data such as sex and smoking were not identified as a significant predictor of clozapine response [34,48,49], despite having a significant effect on clozapine concentrations [12]. However, there were considerable differences to address in terms of clozapine tolerability between males and females [47,49].

This study was limited by available data during the retrospective data collection. Furthermore, due to the limited sample population, all the patients’ data were utilized to develop the model; thus, external validation was not performed and can be a potential topic of future research. However, the internal validation, especially the visual predictive check, is also valuable to investigate the model performance and was utilized in several studies to describe the performance of the models when external validation was not performed [22,50,51]. Information about clozapine concentrations was not available for evaluation as another measure of exposure. However, detailed information about the dose was available and evaluated in different parameterizations, including Emax models, and the cumulative dose after six months was significantly increasing the hazard of symptoms improvement.

## 5. Conclusions

In the current investigation, a repeated time-to-event model was successfully developed and internally validated for positive symptoms improvement after the onset of clozapine treatment among Malaysian SSD patients. Adding a second atypical antipsychotic agent, younger age, and higher cumulative doses of clozapine have predicted a higher hazard of positive symptoms improvement. This model provides valuable information on different hazards of improving positive symptoms after the first clozapine dose administration and modifiable predictors with important implications in clinical practice. The current results support the clozapine treatment longer than 24 weeks even when the targeted response has not yet been achieved, taking into consideration early initiation at a younger age, the augmentation with a second atypical antipsychotic agent, and the adherence to a high maintenance dose, especially in the first six months of treatment.

## Figures and Tables

**Table 1:** Demographic and clinical characteristics of Malaysian patients with schizophrenia spectrum disorders on clozapine (n = 116).

Demographics	Mean ± SD	Clinical Data	Mean ± SD
Age, years	41.4 ± 12.2	Total daily dose, mg	254.2 ± 172.4
Weight, Kg	65.5 ± 15.7	Dose at sex months, mg	259.7 ± 154.4
Height, cm	160.0 ± 5.9	Systolic blood pressure, mmHg	114.0 ± 15.2
BMI, Kg.m^−2^	25.6 ± 5.9	Diastolic blood pressure, mmHg	78.1 ± 11.5
		Pulse rate, beats per minute	94.5 ± 14.3
		White blood cells, 103 cells · µL^−1^	7.7 ± 2.4
		Hemoglobin, g · dL^−1^	13.8 ± 8.0
		Platelet counts, 103 cells · µL^−1^	280.2 ± 65.7
		Neutrophils count, 103 cells · µL^−1^	4.8 ± 2.9
Demographics	n (%)	Clinical data	n (%)
Race		Clozapine once-daily dosing	57 (44.9)
Malay	25 (21.5)	Concurrent medications	
Malaysian Chinese	74 (63.8)	Typical antipsychotic	13 (11.2)
Malaysian Indian	17 (14.7)	Atypical antipsychotic	21 (18.1)
Sex		Anticonvulsants	26 (22.4)
Females	65 (56.0)	Benzodiazepines	8 (6.9)
Males	51 (44.0)	SSRI	26 (22.4)
Smokers	10 (8.6)	Tricyclic antidepressants	8 (6.9)
		Statins	13 (11.2)
		Lactulose	46 (39.7)
		Propranolol	49 (42.2)
		Benzhexol	47 (40.5)

Abbreviations: SD, Standard deviation; SSRI, selective serotonin reuptake inhibitors, BMI, body mass index.

**Table 2:** Objective function value and the number of parameters in different tested survival models.

Number of Parameters	Variable	Model	OFV	ΔOFV	*p*-Value
2	Constant	*h*(*t*) = $\theta_{1}$	1545.99	0	0
3	Gompertz	*h*(*t*) = $\theta_{1}\times e^{\left(\theta_{2}\times t\right)}$	1538.61	7.38	0.0066
3	Weibull	*h*(*t*) = $\theta_{1}\times e^{\left(\theta_{2}\times \ln\left(t\right)\right)}$	1536.55	9.44	0.0021

Abbreviations: OFV, objective function value; *h*, hazard; *t*, time.

**Table 3:** Univariate analysis of covariate effects on the hazard of improving symptoms among Malaysian patients with schizophrenia spectrum disorders (n = 116).

Covariate	ΔOFV	*p*-Value	RSE%	HR	95% CI	IIV% *
Base, no covariate	0	-	-	-	-	126.5
Age	−26.00	<0.0001	25	0.94	0.91–0.97	128.1
Atypical antipsychotics	−17.97	<0.0001	23	5.00	2.42–10.34	110.0
Six months clozapine cumulative dose	−14.29	0.0002	23	1.02	1.01–1.03	113.6
Statin	−5.31	0.0212	43	0.28	0.09–0.82	124.5
Malay race	−3.44	0.0636	56	0.51	0.24–1.07	121.7
Chinese race	−3.25	0.0714	58	1.73	0.93–3.24	122.9
Typical antipsychotics	−2.41	0.1206	58	0.47	0.21–1.11	124.9
Total daily dose after six months	−1.81	0.1785	80	1.00	1.00–1.00	122.9
Benzhexol	−1.44	0.2301	89	0.70	0.37–1.31	130.4
Anticonvulsants	−0.60	0.4386	137	0.76	0.37–1.57	127.7
Indian race	−0.21	0.6468	222	0.82	0.35–1.94	126.5
Smoker	−0.18	0.6714	204	1.23	0.53–2.84	126.5
Sex	−0.12	0.7290	278	1.11	0.64–1.92	126.5
Body mass index	−0.07	0.7913	458	1.01	0.95–1.07	127.3
Once daily dosing	−0.07	0.7913	386	1.07	0.64–1.80	125.3
Clozapine total daily dose	−0.03	0.8625	774	1.00	1.00–1.00	125.7
Benzodiazepines	0.00	1.0000	1335	1.02	0.54–1.93	126.5

Abbreviations: ΔOFV, change in objective function value; RSE, relative standard error; 95% CI, 95% confidence intervals; HR, unadjusted hazard ratio; IIV%, inter-individual variability. * The IIVs shown here are the IIVs for the base model. The IIVs are recorded without and with different covariates addition to the model.

**Table 4:** Stepwise repeated time to improving positive symptoms model building among Malaysian patients with schizophrenia spectrum disorders (n = 116).

N	COV	Model	ΔOFV	*p*-Value
Forward addition				
1	Base	*h*(*t*) = $\theta_{1}\times e^{\left(\theta_{2}\times \ln\left(t\right)\right)}$	0	-
2	Age	*h*(*t*) = $\theta_{1}\times e^{\left(\theta_{2}\times \ln\left(t\right)+\theta_{3}\left(Age-41\right)\right)}$	−25.62	<0.0001
3	AAP	*h*(*t*) = $\theta_{1}\times e^{\left(\theta_{2}\times \ln\left(t\right)+\theta_{3}\left(Age-41\right)+\theta_{4}\left(AAP\right)\right)}$	−10.87	0.0010
4	CTDD	*h*(*t*) = $\theta_{1}\times e^{\left(\theta_{2}\times \ln\left(t\right)+\theta_{3}\left(Age-41\right)+\theta_{4}\left(AAP\right)+\theta_{5}\left(CTDD-34\right)\right)}$	−10.63	0.0011
5	Statin	*h*(*t*) = $\theta_{1}\times e^{\left(\theta_{2}\times \ln\left(t\right)+\theta_{3}\left(Age-41\right)+\theta_{4}\left(AAP\right)+\theta_{5}\left(CTDD-34\right)+\theta_{6}\left(statin\right)\right)}$	−3.52	0.0606
Backward elimination				
6	AAP	*h*(*t*) = $\theta_{1}\times e^{\left(\theta_{2}\times \ln\left(t\right)+\theta_{3}\left(Age-41\right)+\theta_{4}\left(CTDD-34\right)\right)}$	+12.56	0.0004
7	Age	*h*(*t*) = $\theta_{1}\times e^{\left(\theta_{2}\times \ln\left(t\right)+\theta_{3}\left(AAP\right)+\theta_{4}\left(CTDD-34\right)\right)}$	+13.48	0.0002

Abbreviations: COV, covariates; ΔOFV, change in objective function value; AAP, atypical antipsychotics; CTDD, clozapine cumulative dose achieved after six months.

**Table 5:** Estimated parameters of the repeated time-to-event final model for improving positive symptoms following clozapine treatment (n = 116).

Parameter		Typical Value	RSE,%	aHR	95% CI
Scale parameter of Weibull function	*θ*_1_	0.0022	19.8	-	0.0013–0.0031
Shape parameter of Weibull function	*θ*_2_	0.853	8.3	-	0.714–0.992
Age	*θ*_3_	−0.0438	30.5	0.96	0.93–0.98
Concomitant AAP	*θ*_4_	1.39	26.1	4.01	1.97–7.17
CTDD	*θ*_5_	0.0183	28.2	1.02	1.01–1.03
IIV	*η*	101%	35.5	-	-

Abbreviations: RSE, relative standard error; 95% CI, 95% confidence intervals; aHR, adjusted hazard ratio; AAP, atypical antipsychotics; IIV, inter-individual variability; CTDD, clozapine cumulative dose achieved after six months.

## Data Availability

The data that support the findings of this study are available from the corresponding author upon reasonable request.

## Supplementary Materials

The following are available online at https://www.mdpi.com/article/10.3390/pharmaceutics13081121/s1, Figure S1: Final model stratified on concomitant atypical antipsychotic, Figure S2: The final repeated time-to-event model of improving positive symptoms after the onset of clozapine treatment stratified on age groups, Control stream and simulated dataset.
