# Supplementary material for: A Repeated Time-to-Positive Symptoms Improvement among Malaysian Patients with Schizophrenia Spectrum Disorders Treated with Clozapine
Source: Pharmaceutics. 2021 Jul 22;13(8):1121. doi: 10.3390/pharmaceutics13081121 (PMC8401956; doi:10.3390/pharmaceutics13081121)
Supplement: Supplementary file 1 [file pharmaceutics-13-01121-s001.zip › pharmaceutics-1218556-supplementary.pdf]

pharmaceutics

MDPI

Supplementary Materials: A Repeated Time-to-Positive Symptoms Improvement among Malaysian Patients with Schizophrenia Spectrum Disorders Treated with Clozapine

Orwa Albitar, Sabariah Noor Harun, Siti Nor Aizah Ahmad and Siti Maisharah Sheikh Ghadzi

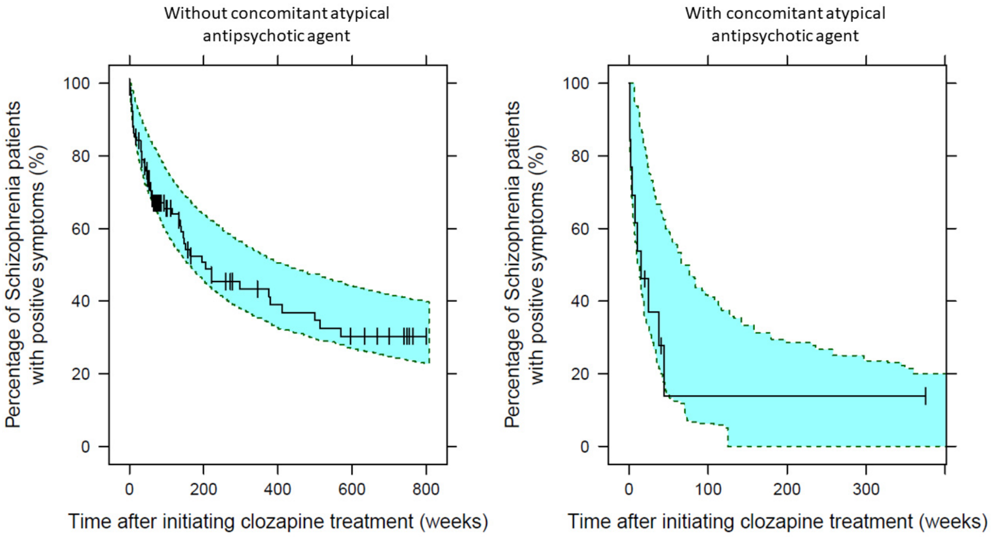

Figure S1. The final repeated time-to-event model of improving positive symptoms after the onset of clozapine treatment stratified on concomitant atypical antipsychotic. The solid line represents the observed Kaplan–Meier survival plot while vertical lines mark censored observations with a mean time, [range] of 306 weeks [8–800]. Shaded areas represent the 95% prediction interval from 1000 simulated datasets. Patients who received a second atypical antipsychotic agent have shown improvement within 50 weeks of clozapine initiation.

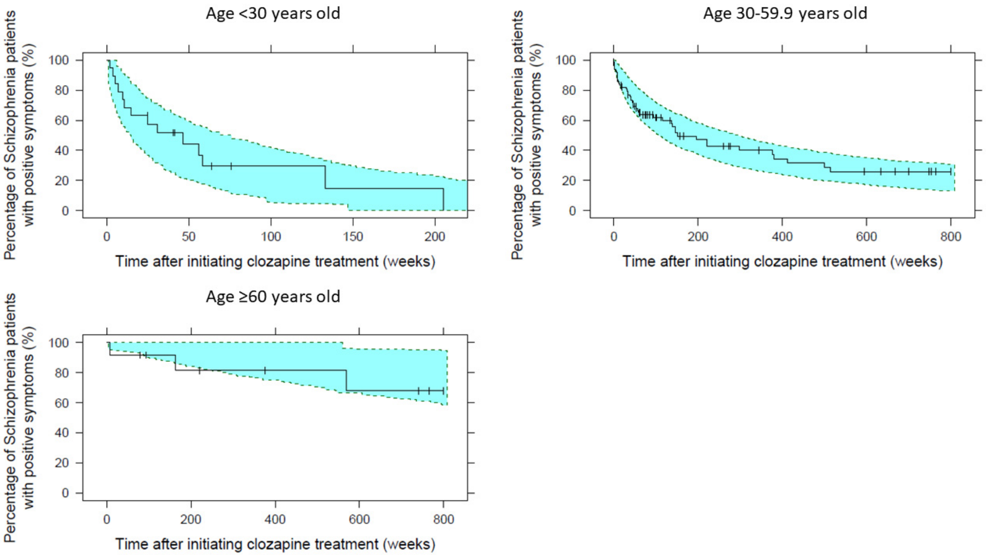

Figure S2. The final repeated time-to-event model of improving positive symptoms after the onset of clozapine treatment stratified on age groups. Young patients less than 30 years old have improved within 200 weeks of clozapine initiation.

Control stream

```
$PROB RTTE CLZ
$INPUT ID TIME DV EVID FLAG AGE CTDD ATA
$DATA simdata.csv IGNORE=@
$SUBROUTINES ADVAN=6 TOL=6
$MODEL COMP=(HAZARD)
$PK
IF(NEWIND.NE.2) TP=0; reset the time of the last event.
LAM = THETA(1)*EXP(ETA(1)); scale parameter of Weibull function
SHP = THETA(2); shape parameter of Weibull function
COVAGE = THETA(3); covariate factor of age
COVATA = THETA(4); covariate factor of concomitant atypical antipsychotics
COVCTDD = THETA(5); covariate factor of cumulative dose at 6 months
$DES
DEL=1E-6; to keep from taking 0**power
DADT(1) = LAM*SHP*(LAM*(T-TP)+DEL)**(SHP-1)*EXP(COVAGE*(AGE-
40.8)+COVATA*ATA+COVCTDD*(CTDD-34.15))
$ERROR
;----------RTTE Model------------------------------
IF(NEWIND.NE.2) OLDCHZ = 0; reset the cumulative hazard
CHZ = A(1) - OLDCHZ; cumulative hazard
OLDCHZ = A(1); rename old cumulative hazard
SUR = EXP(-CHZ); survival probability
DELX = 1E-6
HAZNOW=LAM*SHP*(LAM*(TIME-TP)+DELX)**(SHP-1)*EXP(COVAGE*(AGE-
40.8)+COVATA*ATA+COVCTDD*(CTDD-34.15))
IF(DV.EQ.0) Y=SUR; censored event (prob of survival)
IF(DV.NE.0) Y=SUR*HAZNOW; prob density function of event
IF(FLAG.EQ.2) TP = TIME; for RTTE. TP is time of previous event
;----------------- INITIAL ESTIMATES --------------------------
$THETA (0.0001, 0.002172); LAMBDA
$THETA (0.0001, 0.8526); SHAPE
$THETA -0.043833; COVAGE
$THETA 1.3865; COVATA
$THETA 0.018343; COVCTDD
$OMEGA 1.0236; for RTTE, IIV is needed
$ESTIMATION NOABORT MAXEVAL=0 METHOD=1 LAPLACE LIKE PRINT=1
MSFO=msfb2 SIGL=9 NSIG=3
$COVARIANCE PRINT=E
$TAB ID TIME DV EVID LAM SHP ONEHEADER NOPRINT FILE=sdtab3
```

Simulated data

```
ID,TIME,DV,EVID,FLAG,AGE,CTDD,ATA
1.00,0.00,0.00,3.00,0.00,49.21,26.66,1.00
1.00,800.00,0.00,0.00,8.00,64.51,26.66,1.00
2.00,0.00,0.00,3.00,0.00,24.43,24.97,1.00
2.00,12.00,1.00,0.00,2.00,24.63,24.97,1.00
2.00,28.00,1.00,0.00,2.00,24.93,24.97,1.00
2.00,38.00,1.00,0.00,2.00,25.13,24.97,1.00
2.00,208.00,1.00,0.00,2.00,28.33,24.97,1.00
2.00,286.00,1.00,0.00,2.00,29.83,24.97,1.00
2.00,297.00,1.00,0.00,2.00,30.03,24.97,1.00
2.00,449.00,1.00,0.00,2.00,33.03,24.97,1.00
2.00,509.00,1.00,0.00,2.00,34.13,24.97,1.00
2.00,555.00,1.00,0.00,2.00,35.03,24.97,1.00
```

2.00,800.00,0.00,0.00,8.00,39.73,24.97,1.00
3.00,0.00,0.00,3.00,0.00,56.24,29.66,0.00
3.00,800.00,0.00,0.00,8.00,71.34,29.66,0.00
4.00,0.00,0.00,3.00,0.00,41.84,39.57,0.00
4.00,800.00,0.00,0.00,8.00,56.54,39.57,0.00
5.00,0.00,0.00,3.00,0.00,49.97,30.48,0.00
5.00,800.00,0.00,0.00,8.00,65.27,30.48,0.00
6.00,0.00,0.00,3.00,0.00,49.74,15.16,1.00
6.00,800.00,0.00,0.00,8.00,65.04,15.16,1.00
7.00,0.00,0.00,3.00,0.00,36.39,38.96,0.00
7.00,767.00,1.00,0.00,2.00,50.89,38.96,0.00
7.00,800.00,0.00,0.00,8.00,51.59,38.96,0.00
8.00,0.00,0.00,3.00,0.00,39.51,34.94,0.00
8.00,46.00,1.00,0.00,2.00,40.31,34.94,0.00
8.00,228.00,1.00,0.00,2.00,43.81,34.94,0.00
8.00,800.00,0.00,0.00,8.00,54.81,34.94,0.00
9.00,0.00,0.00,3.00,0.00,26.32,32.68,0.00
9.00,31.00,1.00,0.00,2.00,26.92,32.68,0.00
9.00,52.00,1.00,0.00,2.00,27.32,32.68,0.00
9.00,127.00,1.00,0.00,2.00,28.72,32.68,0.00
9.00,255.00,1.00,0.00,2.00,31.22,32.68,0.00
9.00,608.00,1.00,0.00,2.00,37.92,32.68,0.00
9.00,800.00,0.00,0.00,8.00,41.62,32.68,0.00
10.00,0.00,0.00,3.00,0.00,64.89,32.66,0.00
10.00,12.00,1.00,0.00,2.00,51.49,32.66,0.00
10.00,70.00,1.00,0.00,2.00,52.59,32.66,0.00
10.00,164.00,1.00,0.00,2.00,54.39,32.66,0.00
10.00,800.00,0.00,0.00,8.00,66.59,32.66,0.00
11.00,0.00,0.00,3.00,0.00,37.99,38.41,0.00
11.00,24.00,1.00,0.00,2.00,37.99,38.41,0.00
11.00,44.00,1.00,0.00,2.00,38.39,38.41,0.00
11.00,63.00,1.00,0.00,2.00,38.79,38.41,0.00
11.00,152.00,1.00,0.00,2.00,40.49,38.41,0.00
11.00,594.00,1.00,0.00,2.00,48.99,38.41,0.00
11.00,598.00,1.00,0.00,2.00,48.99,38.41,0.00
11.00,603.00,1.00,0.00,2.00,49.09,38.41,0.00
11.00,800.00,0.00,0.00,8.00,52.89,38.41,0.00
12.00,0.00,0.00,3.00,0.00,31.78,37.97,0.00
12.00,33.00,1.00,0.00,2.00,28.38,37.97,0.00
12.00,199.00,1.00,0.00,2.00,31.58,37.97,0.00
12.00,642.00,1.00,0.00,2.00,40.08,37.97,0.00
12.00,800.00,0.00,0.00,8.00,43.08,37.97,0.00
13.00,0.00,0.00,3.00,0.00,66.44,33.51,0.00
13.00,662.00,1.00,0.00,2.00,65.44,33.51,0.00
13.00,800.00,0.00,0.00,8.00,68.04,33.51,0.00
14.00,0.00,0.00,3.00,0.00,70.41,11.29,0.00
14.00,800.00,0.00,0.00,8.00,59.21,11.29,0.00
15.00,0.00,0.00,3.00,0.00,50.43,35.02,0.00
15.00,167.00,1.00,0.00,2.00,43.53,35.02,0.00
15.00,325.00,1.00,0.00,2.00,46.53,35.02,0.00
15.00,800.00,0.00,0.00,8.00,55.63,35.02,0.00
16.00,0.00,0.00,3.00,0.00,41.29,34.86,0.00
16.00,800.00,0.00,0.00,8.00,56.39,34.86,0.00
17.00,0.00,0.00,3.00,0.00,47.76,37.29,0.00
17.00,800.00,0.00,0.00,8.00,63.06,37.29,0.00

18.00,0.00,0.00,3.00,0.00,28.41,19.79,0.00
18.00,63.00,1.00,0.00,2.00,29.41,19.79,0.00
18.00,364.00,1.00,0.00,2.00,35.21,19.79,0.00
18.00,664.00,1.00,0.00,2.00,40.91,19.79,0.00
18.00,800.00,0.00,0.00,8.00,43.51,19.79,0.00
19.00,0.00,0.00,3.00,0.00,44.81,17.79,0.00
19.00,68.00,1.00,0.00,2.00,46.11,17.79,0.00
19.00,281.00,1.00,0.00,2.00,50.21,17.79,0.00
19.00,383.00,1.00,0.00,2.00,52.21,17.79,0.00
19.00,800.00,0.00,0.00,8.00,60.21,17.79,0.00
20.00,0.00,0.00,3.00,0.00,17.15,60.26,0.00
20.00,74.00,1.00,0.00,2.00,18.45,60.26,0.00
20.00,89.00,1.00,0.00,2.00,18.75,60.26,0.00
20.00,134.00,1.00,0.00,2.00,19.65,60.26,0.00
20.00,664.00,1.00,0.00,2.00,29.75,60.26,0.00
20.00,800.00,0.00,0.00,8.00,32.35,60.26,0.00
21.00,0.00,0.00,3.00,0.00,11.79,52.01,0.00
21.00,16.00,1.00,0.00,2.00,12.09,52.01,0.00
21.00,800.00,0.00,0.00,8.00,27.09,52.01,0.00
22.00,0.00,0.00,3.00,0.00,35.25,57.65,0.00
22.00,66.00,1.00,0.00,2.00,36.55,57.65,0.00
22.00,81.00,1.00,0.00,2.00,36.85,57.65,0.00
22.00,555.00,1.00,0.00,2.00,45.85,57.65,0.00
22.00,800.00,0.00,0.00,8.00,50.55,57.65,0.00
23.00,0.00,0.00,3.00,0.00,35.80,32.70,0.00
23.00,479.00,1.00,0.00,2.00,44.90,32.70,0.00
23.00,624.00,1.00,0.00,2.00,47.70,32.70,0.00
23.00,729.00,1.00,0.00,2.00,49.70,32.70,0.00
23.00,800.00,0.00,0.00,8.00,51.00,32.70,0.00
24.00,0.00,0.00,3.00,0.00,35.51,21.09,0.00
24.00,800.00,0.00,0.00,8.00,50.81,21.09,0.00
25.00,0.00,0.00,3.00,0.00,42.75,7.45,0.00
25.00,800.00,0.00,0.00,8.00,57.65,7.45,0.00
26.00,0.00,0.00,3.00,0.00,49.69,19.01,0.00
26.00,800.00,0.00,0.00,8.00,65.09,19.01,0.00
27.00,0.00,0.00,3.00,0.00,41.47,31.63,0.00
27.00,800.00,0.00,0.00,8.00,56.77,31.63,0.00
28.00,0.00,0.00,3.00,0.00,37.02,35.19,0.00
28.00,325.00,1.00,0.00,2.00,43.22,35.19,0.00
28.00,580.00,1.00,0.00,2.00,48.12,35.19,0.00
28.00,800.00,0.00,0.00,8.00,52.32,35.19,0.00
29.00,0.00,0.00,3.00,0.00,17.76,43.84,0.00
29.00,20.00,1.00,0.00,2.00,18.06,43.84,0.00
29.00,41.00,1.00,0.00,2.00,18.46,43.84,0.00
29.00,187.00,1.00,0.00,2.00,21.26,43.84,0.00
29.00,284.00,1.00,0.00,2.00,23.16,43.84,0.00
29.00,410.00,1.00,0.00,2.00,25.56,43.84,0.00
29.00,438.00,1.00,0.00,2.00,26.16,43.84,0.00
29.00,472.00,1.00,0.00,2.00,26.76,43.84,0.00
29.00,800.00,0.00,0.00,8.00,33.06,43.84,0.00
30.00,0.00,0.00,3.00,0.00,63.01,16.59,0.00
30.00,800.00,0.00,0.00,8.00,78.21,16.59,0.00
31.00,0.00,0.00,3.00,0.00,47.91,44.90,0.00
31.00,94.00,1.00,0.00,2.00,37.01,44.90,0.00
31.00,114.00,1.00,0.00,2.00,37.31,44.90,0.00

31.00,222.00,1.00,0.00,2.00,39.41,44.90,0.00
31.00,362.00,1.00,0.00,2.00,42.11,44.90,0.00
31.00,504.00,1.00,0.00,2.00,44.81,44.90,0.00
31.00,800.00,0.00,0.00,8.00,50.51,44.90,0.00
32.00,0.00,0.00,3.00,0.00,48.13,32.32,0.00
32.00,153.00,1.00,0.00,2.00,50.93,32.32,0.00
32.00,440.00,1.00,0.00,2.00,56.43,32.32,0.00
32.00,800.00,0.00,0.00,8.00,63.33,32.32,0.00
33.00,0.00,0.00,3.00,0.00,32.26,17.84,0.00
33.00,9.00,1.00,0.00,2.00,32.36,17.84,0.00
33.00,22.00,1.00,0.00,2.00,32.56,17.84,0.00
33.00,34.00,1.00,0.00,2.00,32.86,17.84,0.00
33.00,36.00,1.00,0.00,2.00,32.86,17.84,0.00
33.00,182.00,1.00,0.00,2.00,35.66,17.84,0.00
33.00,242.00,1.00,0.00,2.00,36.86,17.84,0.00
33.00,340.00,1.00,0.00,2.00,38.66,17.84,0.00
33.00,393.00,1.00,0.00,2.00,39.76,17.84,0.00
33.00,428.00,1.00,0.00,2.00,40.36,17.84,0.00
33.00,621.00,1.00,0.00,2.00,44.06,17.84,0.00
33.00,800.00,0.00,0.00,8.00,47.56,17.84,0.00
34.00,0.00,0.00,3.00,0.00,41.09,29.61,0.00
34.00,800.00,0.00,0.00,8.00,56.39,29.61,0.00
35.00,0.00,0.00,3.00,0.00,33.34,42.06,0.00
35.00,16.00,1.00,0.00,2.00,34.74,42.06,0.00
35.00,317.00,1.00,0.00,2.00,40.54,42.06,0.00
35.00,352.00,1.00,0.00,2.00,41.24,42.06,0.00
35.00,486.00,1.00,0.00,2.00,43.74,42.06,0.00
35.00,496.00,1.00,0.00,2.00,43.94,42.06,0.00
35.00,800.00,0.00,0.00,8.00,49.74,42.06,0.00
36.00,0.00,0.00,3.00,0.00,53.75,14.76,1.00
36.00,800.00,0.00,0.00,8.00,69.05,14.76,1.00
37.00,0.00,0.00,3.00,0.00,45.09,136.99,0.00
37.00,14.00,1.00,0.00,2.00,45.29,136.99,0.00
37.00,145.00,1.00,0.00,2.00,47.79,136.99,0.00
37.00,216.00,1.00,0.00,2.00,49.19,136.99,0.00
37.00,288.00,1.00,0.00,2.00,50.59,136.99,0.00
37.00,448.00,1.00,0.00,2.00,53.59,136.99,0.00
37.00,623.00,1.00,0.00,2.00,56.99,136.99,0.00
37.00,642.00,1.00,0.00,2.00,57.39,136.99,0.00
37.00,653.00,1.00,0.00,2.00,57.59,136.99,0.00
37.00,739.00,1.00,0.00,2.00,59.19,136.99,0.00
37.00,800.00,0.00,0.00,8.00,60.39,136.99,0.00
38.00,0.00,0.00,3.00,0.00,32.49,3.41,0.00
38.00,800.00,0.00,0.00,8.00,48.09,3.41,0.00
39.00,0.00,0.00,3.00,0.00,37.36,68.34,0.00
39.00,800.00,0.00,0.00,8.00,52.66,68.34,0.00
40.00,0.00,0.00,3.00,0.00,31.04,85.26,0.00
40.00,64.00,1.00,0.00,2.00,31.94,85.26,0.00
40.00,294.00,1.00,0.00,2.00,36.44,85.26,0.00
40.00,406.00,1.00,0.00,2.00,38.54,85.26,0.00
40.00,750.00,1.00,0.00,2.00,45.14,85.26,0.00
40.00,800.00,0.00,0.00,8.00,46.14,85.26,0.00
41.00,0.00,0.00,3.00,0.00,64.47,14.33,0.00
41.00,587.00,1.00,0.00,2.00,75.47,14.33,0.00
41.00,800.00,0.00,0.00,8.00,79.57,14.33,0.00

42.00,0.00,0.00,3.00,0.00,43.44,75.46,0.00
42.00,662.00,1.00,0.00,2.00,55.94,75.46,0.00
42.00,800.00,0.00,0.00,8.00,58.64,75.46,0.00
43.00,0.00,0.00,3.00,0.00,33.39,28.21,0.00
43.00,173.00,1.00,0.00,2.00,36.49,28.21,0.00
43.00,376.00,1.00,0.00,2.00,40.39,28.21,0.00
43.00,548.00,1.00,0.00,2.00,43.69,28.21,0.00
43.00,800.00,0.00,0.00,8.00,48.49,28.21,0.00
44.00,0.00,0.00,3.00,0.00,37.17,31.03,0.00
44.00,800.00,0.00,0.00,8.00,52.47,31.03,0.00
45.00,0.00,0.00,3.00,0.00,26.68,56.92,0.00
45.00,800.00,0.00,0.00,8.00,42.18,56.92,0.00
46.00,0.00,0.00,3.00,0.00,44.79,68.81,0.00
46.00,435.00,1.00,0.00,2.00,50.39,68.81,0.00
46.00,800.00,0.00,0.00,8.00,57.39,68.81,0.00
47.00,0.00,0.00,3.00,0.00,38.99,54.82,0.00
47.00,192.00,1.00,0.00,2.00,42.59,54.82,0.00
47.00,446.00,1.00,0.00,2.00,47.49,54.82,0.00
47.00,800.00,0.00,0.00,8.00,54.29,54.82,0.00
49.00,0.00,0.00,3.00,0.00,25.16,31.44,0.00
49.00,4.00,1.00,0.00,2.00,25.06,31.44,0.00
49.00,9.00,1.00,0.00,2.00,25.16,31.44,0.00
49.00,16.00,1.00,0.00,2.00,25.26,31.44,0.00
49.00,67.00,1.00,0.00,2.00,26.26,31.44,0.00
49.00,93.00,1.00,0.00,2.00,26.76,31.44,0.00
49.00,124.00,1.00,0.00,2.00,27.36,31.44,0.00
49.00,132.00,1.00,0.00,2.00,27.46,31.44,0.00
49.00,179.00,1.00,0.00,2.00,28.36,31.44,0.00
49.00,260.00,1.00,0.00,2.00,29.96,31.44,0.00
49.00,304.00,1.00,0.00,2.00,30.76,31.44,0.00
49.00,311.00,1.00,0.00,2.00,30.96,31.44,0.00
49.00,472.00,1.00,0.00,2.00,34.06,31.44,0.00
49.00,520.00,1.00,0.00,2.00,34.96,31.44,0.00
49.00,618.00,1.00,0.00,2.00,36.86,31.44,0.00
49.00,659.00,1.00,0.00,2.00,37.56,31.44,0.00
49.00,800.00,0.00,0.00,8.00,40.26,31.44,0.00
50.00,0.00,0.00,3.00,0.00,47.77,30.89,0.00
50.00,800.00,0.00,0.00,8.00,63.07,30.89,0.00
51.00,0.00,0.00,3.00,0.00,27.21,41.89,0.00
51.00,409.00,1.00,0.00,2.00,35.01,41.89,0.00
51.00,800.00,0.00,0.00,8.00,42.51,41.89,0.00
52.00,0.00,0.00,3.00,0.00,24.56,46.74,0.00
52.00,21.00,1.00,0.00,2.00,24.66,46.74,0.00
52.00,70.00,1.00,0.00,2.00,25.66,46.74,0.00
52.00,110.00,1.00,0.00,2.00,26.36,46.74,0.00
52.00,111.00,1.00,0.00,2.00,26.36,46.74,0.00
52.00,248.00,1.00,0.00,2.00,29.06,46.74,0.00
52.00,390.00,1.00,0.00,2.00,31.76,46.74,0.00
52.00,467.00,1.00,0.00,2.00,33.26,46.74,0.00
52.00,800.00,0.00,0.00,8.00,39.56,46.74,0.00
53.00,0.00,0.00,3.00,0.00,43.73,38.67,0.00
53.00,124.00,1.00,0.00,2.00,46.03,38.67,0.00
53.00,418.00,1.00,0.00,2.00,51.73,38.67,0.00
53.00,800.00,0.00,0.00,8.00,59.03,38.67,0.00
54.00,0.00,0.00,3.00,0.00,71.97,38.88,0.00

54.00,139.00,1.00,0.00,2.00,74.57,38.88,0.00
54.00,800.00,0.00,0.00,8.00,87.27,38.88,0.00
55.00,0.00,0.00,3.00,0.00,20.54,37.16,1.00
55.00,32.00,1.00,0.00,2.00,21.04,37.16,1.00
55.00,53.00,1.00,0.00,2.00,21.44,37.16,1.00
55.00,140.00,1.00,0.00,2.00,23.14,37.16,1.00
55.00,237.00,1.00,0.00,2.00,24.94,37.16,1.00
55.00,317.00,1.00,0.00,2.00,26.54,37.16,1.00
55.00,325.00,1.00,0.00,2.00,26.64,37.16,1.00
55.00,353.00,1.00,0.00,2.00,27.24,37.16,1.00
55.00,367.00,1.00,0.00,2.00,27.44,37.16,1.00
55.00,384.00,1.00,0.00,2.00,27.84,37.16,1.00
55.00,407.00,1.00,0.00,2.00,28.24,37.16,1.00
55.00,529.00,1.00,0.00,2.00,30.54,37.16,1.00
55.00,532.00,1.00,0.00,2.00,30.64,37.16,1.00
55.00,697.00,1.00,0.00,2.00,33.84,37.16,1.00
55.00,727.00,1.00,0.00,2.00,34.34,37.16,1.00
55.00,734.00,1.00,0.00,2.00,34.54,37.16,1.00
55.00,800.00,0.00,0.00,8.00,35.74,37.16,1.00
56.00,0.00,0.00,3.00,0.00,59.35,34.85,0.00
56.00,222.00,1.00,0.00,2.00,53.05,34.85,0.00
56.00,800.00,0.00,0.00,8.00,64.05,34.85,0.00
57.00,0.00,0.00,3.00,0.00,47.68,43.82,0.00
57.00,800.00,0.00,0.00,8.00,62.38,43.82,0.00
58.00,0.00,0.00,3.00,0.00,37.82,18.48,0.00
58.00,800.00,0.00,0.00,8.00,53.02,18.48,0.00
59.00,0.00,0.00,3.00,0.00,34.44,53.36,0.00
59.00,20.00,1.00,0.00,2.00,34.74,53.36,0.00
59.00,194.00,1.00,0.00,2.00,38.04,53.36,0.00
59.00,420.00,1.00,0.00,2.00,42.34,53.36,0.00
59.00,539.00,1.00,0.00,2.00,44.64,53.36,0.00
59.00,650.00,1.00,0.00,2.00,46.74,53.36,0.00
59.00,800.00,0.00,0.00,8.00,49.64,53.36,0.00
60.00,0.00,0.00,3.00,0.00,60.02,2.38,0.00
60.00,800.00,0.00,0.00,8.00,75.32,2.38,0.00
61.00,0.00,0.00,3.00,0.00,53.46,47.14,0.00
61.00,800.00,0.00,0.00,8.00,68.76,47.14,0.00
62.00,0.00,0.00,3.00,0.00,42.45,63.45,0.00
62.00,436.00,1.00,0.00,2.00,50.75,63.45,0.00
62.00,800.00,0.00,0.00,8.00,57.65,63.45,0.00
63.00,0.00,0.00,3.00,0.00,16.28,20.72,0.00
63.00,394.00,1.00,0.00,2.00,23.88,20.72,0.00
63.00,407.00,1.00,0.00,2.00,24.08,20.72,0.00
63.00,800.00,0.00,0.00,8.00,31.68,20.72,0.00
64.00,0.00,0.00,3.00,0.00,31.06,46.44,0.00
64.00,800.00,0.00,0.00,8.00,46.26,46.44,0.00
65.00,0.00,0.00,3.00,0.00,30.57,116.43,0.00
65.00,3.00,1.00,0.00,2.00,30.67,116.43,0.00
65.00,4.00,1.00,0.00,2.00,30.67,116.43,0.00
65.00,6.00,1.00,0.00,2.00,30.67,116.43,0.00
65.00,13.00,1.00,0.00,2.00,30.87,116.43,0.00
65.00,36.00,1.00,0.00,2.00,31.27,116.43,0.00
65.00,87.00,1.00,0.00,2.00,32.27,116.43,0.00
65.00,136.00,1.00,0.00,2.00,33.17,116.43,0.00
65.00,147.00,1.00,0.00,2.00,33.37,116.43,0.00

65.00,248.00,1.00,0.00,2.00,35.37,116.43,0.00
65.00,266.00,1.00,0.00,2.00,35.67,116.43,0.00
65.00,293.00,1.00,0.00,2.00,36.17,116.43,0.00
65.00,383.00,1.00,0.00,2.00,37.87,116.43,0.00
65.00,449.00,1.00,0.00,2.00,39.17,116.43,0.00
65.00,478.00,1.00,0.00,2.00,39.77,116.43,0.00
65.00,518.00,1.00,0.00,2.00,40.47,116.43,0.00
65.00,546.00,1.00,0.00,2.00,41.07,116.43,0.00
65.00,620.00,1.00,0.00,2.00,42.47,116.43,0.00
65.00,700.00,1.00,0.00,2.00,43.97,116.43,0.00
65.00,707.00,1.00,0.00,2.00,44.17,116.43,0.00
65.00,800.00,0.00,0.00,8.00,45.87,116.43,0.00
66.00,0.00,0.00,3.00,0.00,20.63,11.67,0.00
66.00,714.00,1.00,0.00,2.00,33.73,11.67,0.00
66.00,800.00,0.00,0.00,8.00,35.43,11.67,0.00
67.00,0.00,0.00,3.00,0.00,26.35,59.85,0.00
67.00,1.00,1.00,0.00,2.00,26.35,59.85,0.00
67.00,95.00,1.00,0.00,2.00,28.15,59.85,0.00
67.00,161.00,1.00,0.00,2.00,29.45,59.85,0.00
67.00,204.00,1.00,0.00,2.00,30.25,59.85,0.00
67.00,302.00,1.00,0.00,2.00,32.15,59.85,0.00
67.00,379.00,1.00,0.00,2.00,33.55,59.85,0.00
67.00,468.00,1.00,0.00,2.00,35.25,59.85,0.00
67.00,482.00,1.00,0.00,2.00,35.55,59.85,0.00
67.00,523.00,1.00,0.00,2.00,36.35,59.85,0.00
67.00,532.00,1.00,0.00,2.00,36.55,59.85,0.00
67.00,618.00,1.00,0.00,2.00,38.15,59.85,0.00
67.00,722.00,1.00,0.00,2.00,40.15,59.85,0.00
67.00,800.00,0.00,0.00,8.00,41.65,59.85,0.00
68.00,0.00,0.00,3.00,0.00,19.28,33.82,0.00
68.00,199.00,1.00,0.00,2.00,23.08,33.82,0.00
68.00,603.00,1.00,0.00,2.00,30.78,33.82,0.00
68.00,800.00,0.00,0.00,8.00,34.58,33.82,0.00
69.00,0.00,0.00,3.00,0.00,34.12,34.58,0.00
69.00,27.00,1.00,0.00,2.00,34.52,34.58,0.00
69.00,71.00,1.00,0.00,2.00,35.42,34.58,0.00
69.00,513.00,1.00,0.00,2.00,43.92,34.58,0.00
69.00,699.00,1.00,0.00,2.00,47.52,34.58,0.00
69.00,800.00,0.00,0.00,8.00,49.42,34.58,0.00
70.00,0.00,0.00,3.00,0.00,54.98,51.52,0.00
70.00,42.00,1.00,0.00,2.00,48.48,51.52,0.00
70.00,86.00,1.00,0.00,2.00,49.28,51.52,0.00
70.00,261.00,1.00,0.00,2.00,52.68,51.52,0.00
70.00,800.00,0.00,0.00,8.00,62.98,51.52,0.00
71.00,0.00,0.00,3.00,0.00,31.44,31.86,0.00
71.00,23.00,1.00,0.00,2.00,31.94,31.86,0.00
71.00,110.00,1.00,0.00,2.00,33.64,31.86,0.00
71.00,578.00,1.00,0.00,2.00,42.54,31.86,0.00
71.00,800.00,0.00,0.00,8.00,46.84,31.86,0.00
72.00,0.00,0.00,3.00,0.00,24.14,98.06,1.00
72.00,17.00,1.00,0.00,2.00,20.24,98.06,1.00
72.00,44.00,1.00,0.00,2.00,20.74,98.06,1.00
72.00,62.00,1.00,0.00,2.00,21.14,98.06,1.00
72.00,87.00,1.00,0.00,2.00,21.54,98.06,1.00
72.00,160.00,1.00,0.00,2.00,22.94,98.06,1.00

72.00,177.00,1.00,0.00,2.00,23.34,98.06,1.00
72.00,196.00,1.00,0.00,2.00,23.64,98.06,1.00
72.00,199.00,1.00,0.00,2.00,23.74,98.06,1.00
72.00,220.00,1.00,0.00,2.00,24.14,98.06,1.00
72.00,259.00,1.00,0.00,2.00,24.84,98.06,1.00
72.00,279.00,1.00,0.00,2.00,25.24,98.06,1.00
72.00,340.00,1.00,0.00,2.00,26.44,98.06,1.00
72.00,353.00,1.00,0.00,2.00,26.64,98.06,1.00
72.00,540.00,1.00,0.00,2.00,30.24,98.06,1.00
72.00,546.00,1.00,0.00,2.00,30.34,98.06,1.00
72.00,572.00,1.00,0.00,2.00,30.84,98.06,1.00
72.00,646.00,1.00,0.00,2.00,32.24,98.06,1.00
72.00,747.00,1.00,0.00,2.00,34.24,98.06,1.00
72.00,800.00,0.00,0.00,8.00,35.24,98.06,1.00
73.00,0.00,0.00,3.00,0.00,31.54,47.76,0.00
73.00,21.00,1.00,0.00,2.00,22.44,47.76,0.00
73.00,800.00,0.00,0.00,8.00,37.34,47.76,0.00
75.00,0.00,0.00,3.00,0.00,49.56,30.89,0.00
75.00,594.00,1.00,0.00,2.00,60.96,30.89,0.00
75.00,800.00,0.00,0.00,8.00,64.86,30.89,0.00
76.00,0.00,0.00,3.00,0.00,41.27,88.63,0.00
76.00,40.00,1.00,0.00,2.00,42.07,88.63,0.00
76.00,73.00,1.00,0.00,2.00,42.67,88.63,0.00
76.00,94.00,1.00,0.00,2.00,43.07,88.63,0.00
76.00,135.00,1.00,0.00,2.00,43.87,88.63,0.00
76.00,469.00,1.00,0.00,2.00,50.27,88.63,0.00
76.00,578.00,1.00,0.00,2.00,52.37,88.63,0.00
76.00,755.00,1.00,0.00,2.00,55.77,88.63,0.00
76.00,800.00,0.00,0.00,8.00,56.57,88.63,0.00
77.00,0.00,0.00,3.00,0.00,37.50,39.35,0.00
77.00,190.00,1.00,0.00,2.00,41.10,39.35,0.00
77.00,432.00,1.00,0.00,2.00,45.70,39.35,0.00
77.00,800.00,0.00,0.00,8.00,52.80,39.35,0.00
78.00,0.00,0.00,3.00,0.00,52.57,52.13,0.00
78.00,9.00,1.00,0.00,2.00,52.67,52.13,0.00
78.00,800.00,0.00,0.00,8.00,67.87,52.13,0.00
79.00,0.00,0.00,3.00,0.00,45.65,27.30,0.00
79.00,44.00,1.00,0.00,2.00,46.35,27.30,0.00
79.00,137.00,1.00,0.00,2.00,48.15,27.30,0.00
79.00,208.00,1.00,0.00,2.00,49.45,27.30,0.00
79.00,781.00,1.00,0.00,2.00,60.45,27.30,0.00
79.00,800.00,0.00,0.00,8.00,60.85,27.30,0.00
80.00,0.00,0.00,3.00,0.00,35.58,43.02,0.00
80.00,23.00,1.00,0.00,2.00,29.38,43.02,0.00
80.00,73.00,1.00,0.00,2.00,30.38,43.02,0.00
80.00,800.00,0.00,0.00,8.00,44.28,43.02,0.00
81.00,0.00,0.00,3.00,0.00,44.72,83.78,0.00
81.00,800.00,0.00,0.00,8.00,59.82,83.78,0.00
82.00,0.00,0.00,3.00,0.00,38.98,33.57,0.00
82.00,480.00,1.00,0.00,2.00,48.18,33.57,0.00
82.00,603.00,1.00,0.00,2.00,50.48,33.57,0.00
82.00,800.00,0.00,0.00,8.00,54.28,33.57,0.00
83.00,0.00,0.00,3.00,0.00,65.61,8.79,0.00
83.00,800.00,0.00,0.00,8.00,80.91,8.79,0.00
84.00,0.00,0.00,3.00,0.00,60.16,114.94,0.00

84.00,57.00,1.00,0.00,2.00,61.16,114.94,0.00
84.00,210.00,1.00,0.00,2.00,64.16,114.94,0.00
84.00,218.00,1.00,0.00,2.00,64.26,114.94,0.00
84.00,548.00,1.00,0.00,2.00,70.66,114.94,0.00
84.00,674.00,1.00,0.00,2.00,73.06,114.94,0.00
84.00,800.00,0.00,0.00,8.00,75.46,114.94,0.00
85.00,0.00,0.00,3.00,0.00,39.18,57.43,1.00
85.00,47.00,1.00,0.00,2.00,37.58,57.43,1.00
85.00,53.00,1.00,0.00,2.00,37.68,57.43,1.00
85.00,99.00,1.00,0.00,2.00,38.58,57.43,1.00
85.00,104.00,1.00,0.00,2.00,38.68,57.43,1.00
85.00,178.00,1.00,0.00,2.00,40.08,57.43,1.00
85.00,598.00,1.00,0.00,2.00,48.08,57.43,1.00
85.00,637.00,1.00,0.00,2.00,48.88,57.43,1.00
85.00,681.00,1.00,0.00,2.00,49.68,57.43,1.00
85.00,800.00,0.00,0.00,8.00,51.98,57.43,1.00
86.00,0.00,0.00,3.00,0.00,23.42,43.68,0.00
86.00,258.00,1.00,0.00,2.00,29.02,43.68,0.00
86.00,403.00,1.00,0.00,2.00,31.82,43.68,0.00
86.00,507.00,1.00,0.00,2.00,33.82,43.68,0.00
86.00,679.00,1.00,0.00,2.00,37.12,43.68,0.00
86.00,800.00,0.00,0.00,8.00,39.42,43.68,0.00
87.00,0.00,0.00,3.00,0.00,20.05,51.75,1.00
87.00,6.00,1.00,0.00,2.00,26.65,51.75,1.00
87.00,30.00,1.00,0.00,2.00,27.15,51.75,1.00
87.00,46.00,1.00,0.00,2.00,27.45,51.75,1.00
87.00,49.00,1.00,0.00,2.00,27.55,51.75,1.00
87.00,86.00,1.00,0.00,2.00,28.25,51.75,1.00
87.00,89.00,1.00,0.00,2.00,28.25,51.75,1.00
87.00,113.00,1.00,0.00,2.00,28.75,51.75,1.00
87.00,153.00,1.00,0.00,2.00,29.45,51.75,1.00
87.00,155.00,1.00,0.00,2.00,29.55,51.75,1.00
87.00,165.00,1.00,0.00,2.00,29.75,51.75,1.00
87.00,178.00,1.00,0.00,2.00,29.95,51.75,1.00
87.00,242.00,1.00,0.00,2.00,31.25,51.75,1.00
87.00,248.00,1.00,0.00,2.00,31.35,51.75,1.00
87.00,261.00,1.00,0.00,2.00,31.55,51.75,1.00
87.00,266.00,1.00,0.00,2.00,31.65,51.75,1.00
87.00,268.00,1.00,0.00,2.00,31.75,51.75,1.00
87.00,291.00,1.00,0.00,2.00,32.15,51.75,1.00
87.00,355.00,1.00,0.00,2.00,33.35,51.75,1.00
87.00,389.00,1.00,0.00,2.00,34.05,51.75,1.00
87.00,395.00,1.00,0.00,2.00,34.15,51.75,1.00
87.00,446.00,1.00,0.00,2.00,35.15,51.75,1.00
87.00,450.00,1.00,0.00,2.00,35.15,51.75,1.00
87.00,474.00,1.00,0.00,2.00,35.65,51.75,1.00
87.00,490.00,1.00,0.00,2.00,35.95,51.75,1.00
87.00,558.00,1.00,0.00,2.00,37.25,51.75,1.00
87.00,570.00,1.00,0.00,2.00,37.45,51.75,1.00
87.00,583.00,1.00,0.00,2.00,37.75,51.75,1.00
87.00,593.00,1.00,0.00,2.00,37.95,51.75,1.00
87.00,628.00,1.00,0.00,2.00,38.65,51.75,1.00
87.00,638.00,1.00,0.00,2.00,38.75,51.75,1.00
87.00,656.00,1.00,0.00,2.00,39.15,51.75,1.00
87.00,657.00,1.00,0.00,2.00,39.15,51.75,1.00

87.00,659.00,1.00,0.00,2.00,39.15,51.75,1.00
87.00,660.00,1.00,0.00,2.00,39.25,51.75,1.00
87.00,681.00,1.00,0.00,2.00,39.65,51.75,1.00
87.00,697.00,1.00,0.00,2.00,39.95,51.75,1.00
87.00,706.00,1.00,0.00,2.00,40.05,51.75,1.00
87.00,747.00,1.00,0.00,2.00,40.85,51.75,1.00
87.00,780.00,1.00,0.00,2.00,41.55,51.75,1.00
87.00,782.00,1.00,0.00,2.00,41.55,51.75,1.00
87.00,800.00,0.00,0.00,8.00,41.85,51.75,1.00
88.00,0.00,0.00,3.00,0.00,40.91,32.19,0.00
88.00,800.00,0.00,0.00,8.00,56.31,32.19,0.00
89.00,0.00,0.00,3.00,0.00,42.86,33.24,0.00
89.00,800.00,0.00,0.00,8.00,58.96,33.24,0.00
90.00,0.00,0.00,3.00,0.00,34.38,20.42,0.00
90.00,800.00,0.00,0.00,8.00,49.78,20.42,0.00
91.00,0.00,0.00,3.00,0.00,24.84,23.36,0.00
91.00,60.00,1.00,0.00,2.00,25.94,23.36,0.00
91.00,800.00,0.00,0.00,8.00,40.14,23.36,0.00
92.00,0.00,0.00,3.00,0.00,38.61,51.00,0.00
92.00,10.00,1.00,0.00,2.00,38.71,51.00,0.00
92.00,104.00,1.00,0.00,2.00,40.51,51.00,0.00
92.00,800.00,0.00,0.00,8.00,53.81,51.00,0.00
93.00,0.00,0.00,3.00,0.00,30.79,61.71,0.00
93.00,436.00,1.00,0.00,2.00,39.09,61.71,0.00
93.00,505.00,1.00,0.00,2.00,40.49,61.71,0.00
93.00,643.00,1.00,0.00,2.00,43.09,61.71,0.00
93.00,800.00,0.00,0.00,8.00,46.09,61.71,0.00
94.00,0.00,0.00,3.00,0.00,54.02,20.08,0.00
94.00,800.00,0.00,0.00,8.00,69.22,20.08,0.00
95.00,0.00,0.00,3.00,0.00,33.43,47.46,0.00
95.00,800.00,0.00,0.00,8.00,48.83,47.46,0.00
96.00,0.00,0.00,3.00,0.00,30.55,39.05,0.00
96.00,354.00,1.00,0.00,2.00,37.35,39.05,0.00
96.00,755.00,1.00,0.00,2.00,45.05,39.05,0.00
96.00,800.00,0.00,0.00,8.00,45.85,39.05,0.00
97.00,0.00,0.00,3.00,0.00,51.89,15.11,0.00
97.00,207.00,1.00,0.00,2.00,52.19,15.11,0.00
97.00,642.00,1.00,0.00,2.00,60.59,15.11,0.00
97.00,764.00,1.00,0.00,2.00,62.89,15.11,0.00
97.00,780.00,1.00,0.00,2.00,63.19,15.11,0.00
97.00,800.00,0.00,0.00,8.00,63.59,15.11,0.00
98.00,0.00,0.00,3.00,0.00,28.11,35.49,1.00
98.00,104.00,1.00,0.00,2.00,27.51,35.49,1.00
98.00,137.00,1.00,0.00,2.00,28.11,35.49,1.00
98.00,141.00,1.00,0.00,2.00,28.21,35.49,1.00
98.00,164.00,1.00,0.00,2.00,28.71,35.49,1.00
98.00,176.00,1.00,0.00,2.00,28.91,35.49,1.00
98.00,296.00,1.00,0.00,2.00,31.21,35.49,1.00
98.00,800.00,0.00,0.00,8.00,40.81,35.49,1.00
99.00,0.00,0.00,3.00,0.00,40.22,41.48,0.00
99.00,412.00,1.00,0.00,2.00,46.02,41.48,0.00
99.00,731.00,1.00,0.00,2.00,52.12,41.48,0.00
99.00,800.00,0.00,0.00,8.00,53.42,41.48,0.00
100.00,0.00,0.00,3.00,0.00,48.63,33.17,0.00
100.00,800.00,0.00,0.00,8.00,63.83,33.17,0.00

101.00,0.00,0.00,3.00,0.00,41.89,12.41,0.00
101.00,35.00,1.00,0.00,2.00,42.19,12.41,0.00
101.00,107.00,1.00,0.00,2.00,43.59,12.41,0.00
101.00,414.00,1.00,0.00,2.00,49.49,12.41,0.00
101.00,800.00,0.00,0.00,8.00,56.89,12.41,0.00
102.00,0.00,0.00,3.00,0.00,27.01,75.09,0.00
102.00,12.00,1.00,0.00,2.00,26.91,75.09,0.00
102.00,27.00,1.00,0.00,2.00,27.21,75.09,0.00
102.00,86.00,1.00,0.00,2.00,28.31,75.09,0.00
102.00,173.00,1.00,0.00,2.00,30.01,75.09,0.00
102.00,233.00,1.00,0.00,2.00,31.21,75.09,0.00
102.00,298.00,1.00,0.00,2.00,32.41,75.09,0.00
102.00,472.00,1.00,0.00,2.00,35.71,75.09,0.00
102.00,473.00,1.00,0.00,2.00,35.81,75.09,0.00
102.00,575.00,1.00,0.00,2.00,37.71,75.09,0.00
102.00,800.00,0.00,0.00,8.00,42.01,75.09,0.00
103.00,0.00,0.00,3.00,0.00,49.09,22.91,0.00
103.00,136.00,1.00,0.00,2.00,51.39,22.91,0.00
103.00,800.00,0.00,0.00,8.00,64.19,22.91,0.00
104.00,0.00,0.00,3.00,0.00,12.02,32.48,1.00
104.00,1.00,1.00,0.00,2.00,12.02,32.48,1.00
104.00,2.00,1.00,0.00,2.00,12.02,32.48,1.00
104.00,6.00,1.00,0.00,2.00,12.12,32.48,1.00
104.00,8.00,1.00,0.00,2.00,12.12,32.48,1.00
104.00,12.00,1.00,0.00,2.00,12.22,32.48,1.00
104.00,17.00,1.00,0.00,2.00,12.32,32.48,1.00
104.00,41.00,1.00,0.00,2.00,12.72,32.48,1.00
104.00,42.00,1.00,0.00,2.00,12.82,32.48,1.00
104.00,44.00,1.00,0.00,2.00,12.82,32.48,1.00
104.00,52.00,1.00,0.00,2.00,13.02,32.48,1.00
104.00,57.00,1.00,0.00,2.00,13.12,32.48,1.00
104.00,65.00,1.00,0.00,2.00,13.22,32.48,1.00
104.00,90.00,1.00,0.00,2.00,13.72,32.48,1.00
104.00,105.00,1.00,0.00,2.00,14.02,32.48,1.00
104.00,115.00,1.00,0.00,2.00,14.22,32.48,1.00
104.00,116.00,1.00,0.00,2.00,14.22,32.48,1.00
104.00,118.00,1.00,0.00,2.00,14.22,32.48,1.00
104.00,120.00,1.00,0.00,2.00,14.32,32.48,1.00
104.00,123.00,1.00,0.00,2.00,14.32,32.48,1.00
104.00,140.00,1.00,0.00,2.00,14.62,32.48,1.00
104.00,146.00,1.00,0.00,2.00,14.82,32.48,1.00
104.00,165.00,1.00,0.00,2.00,15.12,32.48,1.00
104.00,172.00,1.00,0.00,2.00,15.32,32.48,1.00
104.00,173.00,1.00,0.00,2.00,15.32,32.48,1.00
104.00,174.00,1.00,0.00,2.00,15.32,32.48,1.00
104.00,176.00,1.00,0.00,2.00,15.32,32.48,1.00
104.00,177.00,1.00,0.00,2.00,15.42,32.48,1.00
104.00,189.00,1.00,0.00,2.00,15.62,32.48,1.00
104.00,195.00,1.00,0.00,2.00,15.72,32.48,1.00
104.00,215.00,1.00,0.00,2.00,16.12,32.48,1.00
104.00,230.00,1.00,0.00,2.00,16.42,32.48,1.00
104.00,241.00,1.00,0.00,2.00,16.62,32.48,1.00
104.00,246.00,1.00,0.00,2.00,16.72,32.48,1.00
104.00,249.00,1.00,0.00,2.00,16.72,32.48,1.00
104.00,271.00,1.00,0.00,2.00,17.22,32.48,1.00

104.00,274.00,1.00,0.00,2.00,17.22,32.48,1.00
104.00,287.00,1.00,0.00,2.00,17.52,32.48,1.00
104.00,292.00,1.00,0.00,2.00,17.62,32.48,1.00
104.00,301.00,1.00,0.00,2.00,17.72,32.48,1.00
104.00,316.00,1.00,0.00,2.00,18.02,32.48,1.00
104.00,319.00,1.00,0.00,2.00,18.12,32.48,1.00
104.00,335.00,1.00,0.00,2.00,18.42,32.48,1.00
104.00,342.00,1.00,0.00,2.00,18.52,32.48,1.00
104.00,363.00,1.00,0.00,2.00,18.92,32.48,1.00
104.00,377.00,1.00,0.00,2.00,19.22,32.48,1.00
104.00,379.00,1.00,0.00,2.00,19.22,32.48,1.00
104.00,389.00,1.00,0.00,2.00,19.42,32.48,1.00
104.00,393.00,1.00,0.00,2.00,19.52,32.48,1.00
104.00,398.00,1.00,0.00,2.00,19.62,32.48,1.00
104.00,410.00,1.00,0.00,2.00,19.82,32.48,1.00
104.00,425.00,1.00,0.00,2.00,20.12,32.48,1.00
104.00,430.00,1.00,0.00,2.00,20.22,32.48,1.00
104.00,455.00,1.00,0.00,2.00,20.72,32.48,1.00
104.00,484.00,1.00,0.00,2.00,21.22,32.48,1.00
104.00,503.00,1.00,0.00,2.00,21.62,32.48,1.00
104.00,530.00,1.00,0.00,2.00,22.12,32.48,1.00
104.00,541.00,1.00,0.00,2.00,22.32,32.48,1.00
104.00,550.00,1.00,0.00,2.00,22.52,32.48,1.00
104.00,570.00,1.00,0.00,2.00,22.92,32.48,1.00
104.00,606.00,1.00,0.00,2.00,23.62,32.48,1.00
104.00,613.00,1.00,0.00,2.00,23.72,32.48,1.00
104.00,638.00,1.00,0.00,2.00,24.22,32.48,1.00
104.00,642.00,1.00,0.00,2.00,24.32,32.48,1.00
104.00,660.00,1.00,0.00,2.00,24.62,32.48,1.00
104.00,669.00,1.00,0.00,2.00,24.82,32.48,1.00
104.00,697.00,1.00,0.00,2.00,25.32,32.48,1.00
104.00,763.00,1.00,0.00,2.00,26.62,32.48,1.00
104.00,800.00,0.00,0.00,8.00,27.32,32.48,1.00
105.00,0.00,0.00,3.00,0.00,47.42,19.39,0.00
105.00,6.00,1.00,0.00,2.00,47.52,19.39,0.00
105.00,85.00,1.00,0.00,2.00,49.02,19.39,0.00
105.00,206.00,1.00,0.00,2.00,51.32,19.39,0.00
105.00,798.00,1.00,0.00,2.00,62.72,19.39,0.00
105.00,800.00,0.00,0.00,8.00,62.72,19.39,0.00
106.00,0.00,0.00,3.00,0.00,51.80,40.80,0.00
106.00,26.00,1.00,0.00,2.00,52.30,40.80,0.00
106.00,136.00,1.00,0.00,2.00,54.40,40.80,0.00
106.00,800.00,0.00,0.00,8.00,67.20,40.80,0.00
107.00,0.00,0.00,3.00,0.00,35.87,33.33,0.00
107.00,38.00,1.00,0.00,2.00,36.57,33.33,0.00
107.00,72.00,1.00,0.00,2.00,37.27,33.33,0.00
107.00,446.00,1.00,0.00,2.00,44.37,33.33,0.00
107.00,800.00,0.00,0.00,8.00,51.17,33.33,0.00
108.00,0.00,0.00,3.00,0.00,51.68,36.57,0.00
108.00,800.00,0.00,0.00,8.00,66.68,36.57,0.00
109.00,0.00,0.00,3.00,0.00,49.56,24.99,0.00
109.00,232.00,1.00,0.00,2.00,53.96,24.99,0.00
109.00,800.00,0.00,0.00,8.00,64.86,24.99,0.00
110.00,0.00,0.00,3.00,0.00,27.83,31.87,0.00
110.00,59.00,1.00,0.00,2.00,16.43,31.87,0.00

110.00,800.00,0.00,0.00,8.00,30.63,31.87,0.00
111.00,0.00,0.00,3.00,0.00,32.67,31.28,0.00
111.00,47.00,1.00,0.00,2.00,33.17,31.28,0.00
111.00,79.00,1.00,0.00,2.00,33.77,31.28,0.00
111.00,213.00,1.00,0.00,2.00,36.37,31.28,0.00
111.00,385.00,1.00,0.00,2.00,39.67,31.28,0.00
111.00,692.00,1.00,0.00,2.00,45.57,31.28,0.00
111.00,800.00,0.00,0.00,8.00,47.67,31.28,0.00
112.00,0.00,0.00,3.00,0.00,42.82,18.68,0.00
112.00,500.00,1.00,0.00,2.00,52.42,18.68,0.00
112.00,773.00,1.00,0.00,2.00,57.62,18.68,0.00
112.00,800.00,0.00,0.00,8.00,58.12,18.68,0.00
113.00,0.00,0.00,3.00,0.00,24.16,14.64,0.00
113.00,192.00,1.00,0.00,2.00,24.56,14.64,0.00
113.00,800.00,0.00,0.00,8.00,36.26,14.64,0.00
114.00,0.00,0.00,3.00,0.00,23.87,27.93,0.00
114.00,32.00,1.00,0.00,2.00,24.07,27.93,0.00
114.00,43.00,1.00,0.00,2.00,24.27,27.93,0.00
114.00,477.00,1.00,0.00,2.00,32.57,27.93,0.00
114.00,800.00,0.00,0.00,8.00,38.77,27.93,0.00
115.00,0.00,0.00,3.00,0.00,40.15,17.15,1.00
115.00,352.00,1.00,0.00,2.00,46.35,17.15,1.00
115.00,354.00,1.00,0.00,2.00,46.35,17.15,1.00
115.00,422.00,1.00,0.00,2.00,47.65,17.15,1.00
115.00,749.00,1.00,0.00,2.00,53.95,17.15,1.00
115.00,800.00,0.00,0.00,8.00,54.95,17.15,1.00
116.00,0.00,0.00,3.00,0.00,21.67,37.63,0.00
116.00,800.00,0.00,0.00,8.00,36.97,37.63,0.00
117.00,0.00,0.00,3.00,0.00,61.00,20.20,0.00
117.00,800.00,0.00,0.00,8.00,76.00,20.20,0.00
118.00,0.00,0.00,3.00,0.00,48.66,45.44,0.00
118.00,11.00,1.00,0.00,2.00,41.76,45.44,0.00
118.00,315.00,1.00,0.00,2.00,47.56,45.44,0.00
118.00,800.00,0.00,0.00,8.00,56.86,45.44,0.00
